# CNN-Based Multi-Modal Camera Model Identification on Video Sequences

**DOI:** 10.3390/jimaging7080135

**Published:** 2021-08-05

**Authors:** Davide Dal Cortivo, Sara Mandelli, Paolo Bestagini, Stefano Tubaro

**Affiliations:** Dipartimento di Elettronica, Informazione e Bioingegneria, Politecnico di Milano, 20133 Milan, Italy; davide.dalcortivo@mail.polimi.it (D.D.C.); paolo.bestagini@polimi.it (P.B.); stefano.tubaro@polimi.it (S.T.)

**Keywords:** camera model identification, video forensics, audio forensics, convolutional neural networks

## Abstract

Identifying the source camera of images and videos has gained significant importance in multimedia forensics. It allows tracing back data to their creator, thus enabling to solve copyright infringement cases and expose the authors of hideous crimes. In this paper, we focus on the problem of camera model identification for video sequences, that is, given a video under analysis, detecting the camera model used for its acquisition. To this purpose, we develop two different CNN-based camera model identification methods, working in a novel multi-modal scenario. Differently from mono-modal methods, which use only the visual or audio information from the investigated video to tackle the identification task, the proposed multi-modal methods jointly exploit audio and visual information. We test our proposed methodologies on the well-known Vision dataset, which collects almost 2000 video sequences belonging to different devices. Experiments are performed, considering native videos directly acquired by their acquisition devices and videos uploaded on social media platforms, such as YouTube and WhatsApp. The achieved results show that the proposed multi-modal approaches significantly outperform their mono-modal counterparts, representing a valuable strategy for the tackled problem and opening future research to even more challenging scenarios.

## 1. Introduction

Camera model identification has gained significant importance in multimedia forensic investigations as digital multimedia contents (i.e., digital images, videos and audio sequences) are increasingly widespread and will continue to spread in the future with the advance of technological progress. This phenomenon is mainly attributable to the advent of the internet and social media, which have allowed a very rapid diffusion of digital contents and, consequently, made it extremely difficult to trace their origin.

For instance, in forensic investigations, tracing the origin of digital contents can be essential to identify the perpetrators of such crimes as rape, drug trafficking or acts of terrorism. There is also the possibility that certain private content become viral through the internet, as has sadly happened in recent times with revenge porn. Being able to retrieve the source of multimedia content, therefore, assumes a fundamental role.

This paper aims at determining the smartphone model used to acquire digital video sequences by jointly exploiting visual and audio information from the videos themselves. We mainly focus on video source identification because little work has been done specifically for digital video sequences in the forensic literature [1]. On the contrary, the analysis of digital images is widely addressed [2]. We can identify the camera model used to acquire an image, thanks to the various peculiar traces left on the photograph at the time of shooting. In this vein, the two main families of approaches related to image camera model identification are defined as model-based and data-driven.

Model-based approaches specifically rely on exploiting the traces released by the digital image acquisition process in order to identify the camera model. Several works in the literature exploit specific features associated with the Color Filter Array (CFA) configuration (i.e., the specific arrangement of color filters in the sensor plane) [3,4] and the CFA interpolation algorithm [5,6,7,8,9] to retrieve information about the source camera model. Undesired optical aberration effects generated by the lens are exploited as well in [10,11,12,13,14]. Moreover, other processing operations and defects (typical of the image acquisition pipeline), such as dust particles left on the sensor [15] and noise patterns [16], have been demonstrated to carry information about the used camera model.

In the last few years, the availability of digital data and computational resources has lead to the growth of data-driven approaches, which have greatly outperformed many model-based solutions proposed in the past. Instead of focusing on a specific trace left by the image acquisition process, as is typically done in model-based methodologies, data-driven approaches are able to capture model traces, due to various components’ interplay [2]. The most recent and best-performing data-driven methodologies are those based on learned features, that is, methods directly feeding digital images to a deep-learning paradigm in order to learn model-related features and to associate images with their original source. In this field, Convolutional Neural Networks (CNNs) are now the most widespread solution [17,18,19,20,21,22].

To our knowledge, the only work that investigates the problem of camera model identification on video sequences is proposed in [1]. The authors exploit a CNN to produce camera model identification scores for small patches extracted from video frames, and then fuse the achieved scores to produce a single accurate classification result per video.

In this paper, we rely on advanced deep-learning approaches to develop effective methods for camera model identification on video sequences. Specifically, our proposed method involves the use of CNNs capable of classifying videos by jointly extracting suitable features from their visual and audio content. We define the proposed strategy as multi-modal since we exploit both visual and audio information coming from the query video to solve the identification task. Given a video, as visual content, we use patches cropped from the frames; as audio content, we use patches cropped from the Log-Mel Spectrogram (LMS) of its audio track. In this vein, the approach suggested by [1] falls into the mono-modal category, as the authors only exploit the visual content to classify a query video.

We propose two distinct multi-modal camera model identification approaches. In both proposed approaches, we make use of CNNs and feed them with pairs of visual and audio patches. In the first approach, we compare and fuse the scores individually obtained from two CNNs, trained following a mono-modal strategy, i.e., one CNN only deals with visual information and the other one only with audio. In the second approach, we train a single multi-input CNN, which deals with both visual and audio patches. Moreover, for each proposed approach, we investigate three different network configurations and data pre-processings, exploiting effective CNN architectures that are well known in the state of the art [23,24].

We evaluate results on the Vision dataset, which contains approximately 650 native video sequences with their related social media versions, collecting almost 2000 videos recorded by 35 modern smartphones. The videos on which we conduct experiments are not only the original native ones; we also use those compressed by the WhatsApp and YouTube algorithms so as to explore the effects of data recompression and to investigate challenging scenarios in which the training and testing datasets do not share common characteristics.

To provide a baseline strategy for comparing the achieved results, we investigate the mono-modal attribution problems as well. Indeed, the vast majority of state-of-the-art works in multimedia forensics always deal with video sequences by only exploiting their visual or audio content in a separate way [25,26,27,28,29]. Only a few works have been proposed that employ both visual and audio cues for multimedia forensics purposes, but they do not tackle the camera model identification task [30,31,32,33]. We evaluate the mono-modal results achieved by exploiting only visual or audio patches to classify the query video sequence. The performed experimental campaign highlights the effectiveness of the proposed multi-modal methodology with respect to mono-modal strategies. In general, the pursued multi-modal approaches demonstrate to be significantly more effective than standard mono-modal solutions. As expected, we verify that data that undergo stronger compression (e.g., videos uploaded to the WhatsApp application) are more challenging to classify. Nonetheless, the proposed multi-modal methods outperform the mono-modal strategies also in this complicated scenario.

Our work is organized as follows. In Section 2, we introduce some general concepts in order to better understand the tackled problem and the proposed methodology. In Section 3, we report the formulation of the problems of mono-modal and multi-modal camera model attribution. In Section 4, we report a detailed explanation of the resolution method proposed. In Section 5, we analyze the achieved results. Finally, Section 6 draws some conclusions.

## 2. Background

Identifying the camera model used to acquire an image or a video frame is possible, thanks to the many peculiar traces left on them at the shooting time. To better understand the traces that we are referring to, in this section, we provide the reader with some background on the generic acquisition pipeline of digital images. Then, since we investigate also the audio content of video sequences, we introduce the definition of the Mel scale and LMS of digital audio signals. In particular, the LMS is a very powerful tool for analyzing the spectral and temporal evolution of an audio track.

### 2.1. Digital Image Acquisition Pipeline

**Figure 1:**
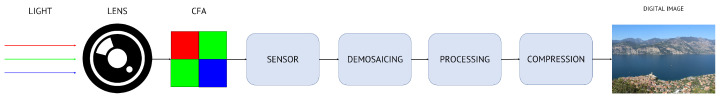
Typical steps of a common digital image acquisition pipeline.

Whenever we take a photograph with a digital camera or smartphone, we trigger an elaborate process consisting of several operations. This process, which lasts a few fractions of a second, starts when we press the shutter button and ends when we can visualize the shot taken. In general, the acquisition pipeline of a digital image is not unique. There can be differences among the vendors, the device models and the on-board technologies that are available. Nonetheless, we can reasonably model the image acquisition pipeline as a series of common steps [34], as depicted in Figure 1.

Light rays hit a lens that focuses them on the sensor [35]. The surface of a sensor is covered by a grid of microscopic pits called photosites, which represent the pixels of a digital image and return a different voltage depending on the intensity of the light that hits them. To capture colors, most sensors use color filters. The most common one is the CFA (or Bayer filter), which covers each photosite with a colored filter (red, green or blue), specializing it in capturing that particular color. The shape of the CFA determines the color captured by each pixel, and this is a vendor choice. Beyond the CFA grid, we end up with three partially sampled color layers, where only one color (i.e., red, blue or green) is impressed at each pixel location. To retrieve the missing color information (e.g., blue and red for pixels that only acquired green light), an interpolation is made between the color captured by the photosite itself and the colors captured by the neighboring photosites. This procedure, namely the demosaicing, debayering or CFA interpolation process, allows to obtain a raw version of color images and can be implemented using proprietary interpolation techniques.

After that, we have a processing phase consisting of additional operations. For instance, as lenses may introduce various kinds of optical aberrations (e.g., radial lens distortion, chromatic aberration, and vignetting), camera vendors typically apply some digital correction; this may introduce forensic traces. Furthermore, other common operations that are vendor-specific are the white balancing and the color correction. Eventually, a step of image compression is typically applied. In this regard, JPEG compression is the most widespread operation and again introduces implementation-specific and quality degrees of freedom.

### 2.2. Mel Scale and Log-Mel Spectrogram

The Mel scale is a perceptual scale of pitches proposed in 1940 by [36]. In particular, the Mel scale aims at mimicking the non-linear human ear perception of sound by being more discriminative at lower frequencies and less discriminative at higher frequencies. The relation between pitch (in Mel scale) and frequency (in Hz) is as follows:

(1) $$p=\mathrm{Mel}\left(f\right)=2595\cdot \log\left(1+\frac{f}{700}\right),$$

where p=Mel(f) indicates the perceived pitch p[Mel] of a sound at frequency f[Hz]. Conversely, we can define as $f={\mathrm{Mel}}^{-1}\left(p\right)$ the inverse relationship, by means of which we can compute the frequency (Hz) starting from the pitch (Mel).

The human ear’s behavior can be simulated with the so-called Mel filterbank, a set of *K* triangular filters, where each filter has a maximum response at the center frequency and decreases linearly toward 0 until it reaches the center frequency of the two adjacent ones. Specifically, the filter centered around the pitch *p* in Mel scale can be modeled as follows:

(2) $$H_{p}\left(f\right)=\left\{\begin{array}{cc} \frac{f-{\mathrm{Mel}}^{-1}\left(p-1\right)}{{\mathrm{Mel}}^{-1}\left(p\right)-{\mathrm{Mel}}^{-1}\left(p-1\right)}, & {\mathrm{Mel}}^{-1}\left(p-1\right)\leq f<{\mathrm{Mel}}^{-1}\left(p\right)\\ \frac{{\mathrm{Mel}}^{-1}\left(p+1\right)-f}{{\mathrm{Mel}}^{-1}\left(p+1\right)-{\mathrm{Mel}}^{-1}\left(p\right)}, & {\mathrm{Mel}}^{-1}\left(p\right)\leq f\leq {\mathrm{Mel}}^{-1}\left(p+1\right)\\ 0, & otherwise \end{array}\right..$$

The entire Mel filterbank can be modeled as a two-dimensional matrix H with size F×K, where columns contain the coefficients associated with the different filters $H_{p}\left(f\right)$ (related to *K* distinct pitches), and rows are associated with frequencies.

By applying the Mel filterbank H to the spectrogram of an audio signal, we can compute the LMS, which is an important tool widely used for speech and audio processing [24,37,38]. Considering a signal evaluated over *T* temporal samples and *F* frequency bins, LMS can be represented as a 2D matrix L with size T×K, computed as follows:

(3) L=ln(S·H+ϵ).

where S is a 2D matrix with size T×F containing the spectrogram of the audio signal (i.e., the magnitude of the Short-Time Fourier Transform (STFT), with frequency information along columns and time information along rows), computes the matrix multiplication, ln(·) computes the natural logarithm, and ϵ is a small constant used to avoid feeding zeros to the logarithm. The resulting LMS brings information about the spectral content of the audio signal (in Mel scale) as a function of the temporal evolution: along columns, we find pitches in Mel scale; along rows, the temporal evolution.

## 3. Problem Formulation

The problem we address in this paper is that of camera model identification on video sequences. We mainly focus on identifying the source camera model of digital video sequences, as the analysis of digital images has been widely addressed in the forensic literature, with excellent results [2,18,21,22]. In particular, we work with video sequences recorded from different smartphone models and propose an innovative approach that combines visual and audio information of the considered videos. In the following sections, we first introduce the standard mono-modal problem, which aims at identifying the source camera model of a video sequence, exploiting only its visual or audio information. Then, we introduce the actual multi-modal problem tackled in this paper, which employs both visual and audio cues to identify the source camera model from videos.

### 3.1. Mono-Modal Camera Model Identification

The problem of mono-modal camera model identification consists of detecting the device model used to acquire a specific kind of media at a single modality, for instance, given a photograph, understanding the model of the camera used to take it, or, alternatively, given an audio recording, detecting the used recorder model. Given a video, which is the case of our interest, the mono-modal model attribution consists of identifying the device model that shot it, using only the visual or audio information of the video itself.

### 3.2. Multi-Modal Camera Model Identification

Given a video sequence, the problem of multi-modal camera model identification converts to identifying the device model that shot it, using both the visual and audio information of the video itself. In our case, we consider a closed-set identification, which consists of detecting the camera model used to shoot a video sequence within a set of known devices. In this scenario, the investigator assumes that the video being analyzed is taken with a device belonging to a family of devices that she/he knows. If the video does not come from any of those devices, the investigator will wrongly attribute the video to one of those.

## 4. Methodology

**Figure 2:**
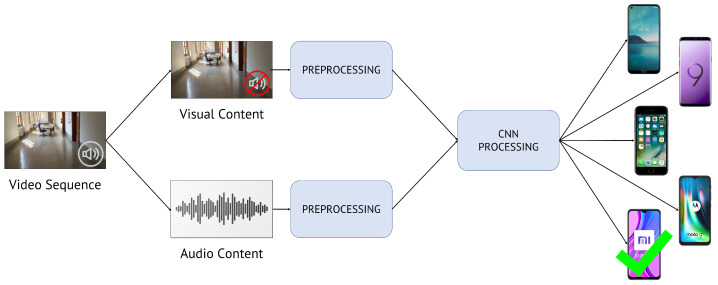
Pipeline of the proposed method to solve multi-modal camera model identification on video sequences. Given a query video sequence, we extract and pre-process its visual and audio content, then feed these data to CNNs in order to identify the actual source camera model.

In this paper, we propose to solve the problem of closed-set multi-modal camera model identification on video sequences. Figure 2 represents the general scheme of the proposed methodology. Starting from the video under analysis, we jointly exploit its visual and audio content to retrieve the smartphone model used to shoot it. In particular, we extract both visual and audio cues of query video sequences and feed these data into one or multiple CNNs that can discriminate among different source camera models. In a nutshell, the proposed method includes two main steps:

1. Content extraction and pre-processing: extraction of visual and audio content from the video sequence under analysis and manipulation of data prior to feeding them to CNNs;
2. CNN processing: feature extraction and classification block composed of one or multiple CNNs.

In the following lines, we enter more in detail for each step of the proposed pipeline.

### 4.1. Content Extraction and Pre-Processing

The extraction and pre-processing phase consists of visual and audio content manipulation and data normalization.

**Figure 3:**
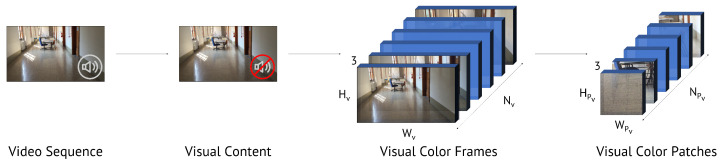
Extraction of visual patches from a video sequence. We extract $N_{v}$ color frames, with size $H_{v}$ and $W_{v}$. From these frames, we randomly extract $N_{P_{v}}$ visual patches with size $H_{P_{v}}\times W_{P_{v}}$.

Considering the extraction and pre-processing of visual content from the video under analysis, this phase consists of three steps (see Figure 3):

1. Extraction of $N_{v}$ color frames equally distant in time and distributed over its entire duration. The video frames have size $H_{v}\times W_{v}$, which depends on the resolution of the video under analysis;
2. Random extraction of $N_{P_{v}}$ color patches of size $H_{P_{v}}\times W_{P_{v}}$;
3. Patch normalization in order to have zero mean and unitary variance as is commonly done prior to feeding data to CNNs.

**Figure 4:**
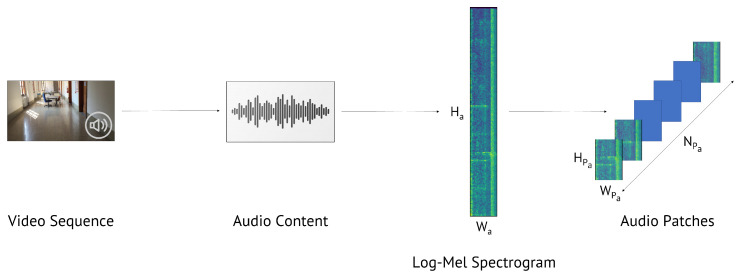
Extraction of audio patches from a video sequence. Once we select the audio content, we compute the LMS, which has size $H_{a}\times W_{a}$. Then, we randomly extract $N_{P_{a}}$ audio patches with size $H_{P_{a}}\times W_{P_{a}}$.

Regarding the audio content of the video under analysis, the extraction and pre-processing phase consists of three steps as well (see Figure 4):

1. Extraction of the LMS L of the audio content related to the video sequence. Indeed, the LMS represents a very informative tool for audio data and was used several times as a valuable feature for audio and speech classification and processing [24,37,38,39,40,41]. During some preliminary experiments, we compared different audio features extracted from the magnitude and phase of the signal STFT, and we verified that the LMS (based on the STFT magnitude) was the most informative one. Phase-based strategies reported accuracy of lower than 80%, achieved by LMS. The LMS L has size $H_{a}\times W_{a}$, where rows refer to the temporal information (varying with the video length) and columns to the frequency content in Mel scale;
2. Random extraction of $N_{P_{a}}$ patches of size $H_{P_{a}}\times W_{P_{a}}$ from L;
3. Patch normalization in order to have zero mean and unitary variance, as previously described for visual patches.

### 4.2. CNN Processing

In the CNN processing step, the extracted pre-processed content is fed to one or multiple CNNs to extract distinguishable features among the different source camera models and classify the original one.

The mono-modal camera identification problem can be solved by feeding the visual or audio information extracted as shown in Section 4.1 to a CNN. In principle, any CNN architecture performing classification could be used at this point; in the next section, we comment our choice in detail. The final layer of the classification network is a fully-connected layer with a number of nodes equal to the total number of models, *M*, where each node is associated with a particular camera model. The output value is an *M*-element vector defined as y, where each element $y_{m}$ represents the probability that input data have been acquired by the model associated with that node. To extract the predicted model $\hat{m}$ in the classification process, we can select the node associated with the maximum score obtained:

(4) $$\hat{m}=\underset{m}{\mathrm{argmax}}y_{m}.$$

Considering multi-modal camera model identification, which is our actual task, we propose two distinct methods to solve the problem:

1. Late Fusion methodology: compare the classification scores of visual and audio patches, separately obtained from two single-input CNNs;
2. Early Fusion methodology: build one multi-input CNN, feed this with both visual and audio content and exploit it to produce a single classification score.

In both proposed methods, we always provide pairs of patches as input to the network(s), composed of one visual patch and one audio patch extracted from the same video sequence under analysis.

#### 4.2.1. Late Fusion Methodology

In the first method, defined as Late Fusion methodology, we follow three steps to determine the predicted model $\hat{m}$ for a visual/audio patch pair coming from the same query video sequence:

1. Separately feed a CNN with a visual patch and a CNN with an audio patch;
2. Extract the classification scores associated with the two patches. In particular, we define $y_{v}$ as the classification scores related to the visual patch and $y_{a}$ as those related to the audio patch;
3. Select the classification score vector (choosing between $y_{v}$ and $y_{a}$) that contains the highest score; the estimated source model $\hat{m}$ by the Late Fusion methodology is related to the position in which that score is found:
  (5) $$\hat{m}=\underset{m}{\mathrm{argmax}}\;y_{{\mathrm{LF}}_{m}},$$
  where $y_{{\mathrm{LF}}_{m}}$ is the *m*-th element of the score vector $y_{\mathrm{LF}}$, defined as follows:
  (6) $$y_{\mathrm{LF}}=\left\{\begin{array}{cc} y_{v} & \mathrm{if}\;\max_{m}\;y_{v_{m}}\geq \max_{m}\;y_{a_{m}}\\ y_{a} & \mathrm{if}\;\max_{m}\;y_{v_{m}}<\max_{m}\;y_{a_{m}} \end{array}\right..$$

**Figure 5:**
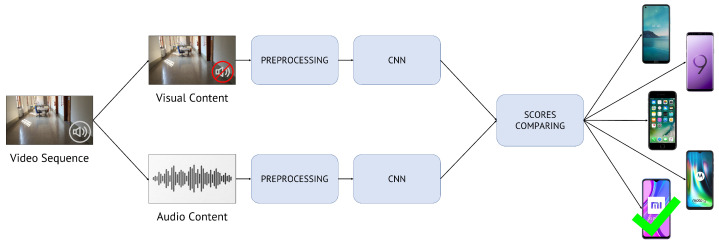
Late Fusion method pipeline. Given a query video, we extract and pre-process its visual and audio content. Then, we separately feed two distinct CNNs: one only with visual information and the other one only with audio information. Eventually, we compare and fuse the classification scores to identify the actual source camera model.

To summarize, Figure 5 depicts the pipeline of the proposed Late Fusion method.

The training phase of Late Fusion method consists of training the two networks (one dealing with visual patches and the other one with audio patches) separately. More specifically, the network working with visual patches updates its weights by optimizing the classification problem on the scores returned by $y_{v}$; the network working with audio patches is optimized basing on the scores returned by $y_{a}$. The two networks are separately trained following the very same mono-modal methodology seen at the beginning of Section 4.2. In the evaluation phase, the results obtained from the two CNN branches are compared and fused.

#### 4.2.2. Early Fusion Methodology

**Figure 6:**
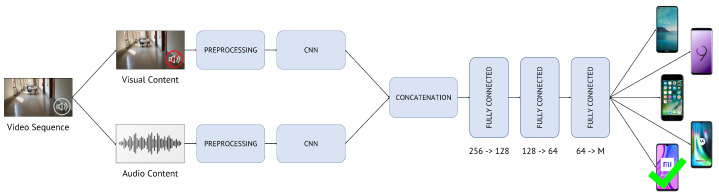
Early Fusion method pipeline. Given a query video, we extract and pre-process its visual and audio content. Then, we feed these data to one multi-input CNN, composed of two CNNs whose last fully-connected layers are concatenated. Three additional fully-connected layers follow to identify the actual source camera model.

In the second method, defined as Early Fusion, we build a multi-input CNN by joining together two CNNs. The union is made by concatenating the final fully-connected layers of the two networks and by adding three fully-connected layers up to the prediction of the camera model (see Figure 6 for details about the layers dimensionality). For each visual/audio patch pair, Early Fusion predicts the estimated camera model based on the scores obtained at the output of the last fully-connected layer, namely $y_{\mathrm{EF}}$:

(7) $$\hat{m}=\underset{m}{\mathrm{argmax}}y_{{\mathrm{EF}}_{m}}.$$

In the training phase, we train the whole network in its entirety using visual and audio patch pairs. Unlike Late Fusion, there is no separate training between the visual and audio branches. Both training and testing phases are analogous to those of the mono-modal methodology, but this time, we provide the whole network with visual/audio patch pairs, not single patches only (e.g., limited to visual or audio content). Figure 6 draws the pipeline of the Early Fusion method. The dimensions of input and output features to the fully-connected layers are reported as well. Notice that the output feature at the last network layer has size equal to *M*, i.e., the number of investigated camera models.

### 4.3. CNN Architectures

The CNNs we use to solve the problem are the EfficientNetB0 [23] and the VGGish [24].

The EfficientNetB0 belongs to the recently proposed EfficientNet family of CNN models [23], which has achieved very good results in multimedia forensics tasks [21]. It is the simplest EfficientNet model; we selected this in order to have faster training phases and, consequently, much more time to experiment with different evaluation setups. Moreover, as shown in [21] and verified by means of preliminary tests, there is no significant difference between the performance of EfficientNetB0 with respect to computationally heavier network models requiring more parameters. The VGGish [24] is a CNN widely used for audio classification [42], and it is inspired by the famous VGG networks [43] used for image classification.

We use EfficientNetB0 for processing visual patches; audio patches can be processed by means of both EfficientNetB0 and VGGish. To solve the proposed multi-modal camera model identification problem, we make some modifications to the network architectures in order to match the input audio data. In particular, to correctly process audio patches, we add an extra convolutional layer at the beginning of EfficientNetB0. We need this additional layer because EfficientNetB0 accepts three-channel patches as input (i.e., color patches). The extra layer applies a 2D convolution using 3×3×3 kernels, resulting in a transformed color patch suitable for EfficientNetB0.

## 5. Results

In this section, we first present the dataset we work with, and the experimental setup (i.e., the network training parameters and the configurations we use in the experiments). Then, we report the evaluation metrics and comment on the achieved results.

### 5.1. Dataset

We select video sequences from the Vision dataset [44], a recent image and video dataset, purposely designed for multimedia forensics investigations. The Vision dataset collects approximately 650 native video sequences captured by 35 modern smartphones/tablets, including also their related social media versions. Overall, the dataset comprises almost 2000 video sequences, clearly indicating the source device used to acquire them. To perform our experiments, we select non-flat videos (i.e., videos depicting natural scenes containing objects): both the original native ones (i.e., videos acquired by the smartphone camera without any post-processing) and those compressed by WhatsApp and YouTube algorithms. Since our analysis is aimed at the granularity model-level, we group videos from different devices that belong to the same model. Videos from devices D04, D12, D17 and D22 (considering the Vision dataset nomenclature provided in [44]) are excluded because they give problems in the extraction of the frames or the audio track. We also exclude the original videos that do not feature a WhatsApp or YouTube compressed version. Notice that we do not limit our investigations to high resolution videos: even though the majority of native videos presents resolutions higher than or equal to 720p, we also explore native sequences limited to 640×480. In doing so, we end up with 1110 videos of about 1 min, belonging to 25 different camera models. For each video sequence, we exploit the provided information about its source camera model as the ground truth to evaluate the classification performance of our proposed method.

For what concerns the visual content of videos, we extract 50 frames per video sequence, equally distant in time and distributed over its entire duration. Then, we extract 10 patches per frame (taken in random positions), for a total of $N_{P_{v}}=500$ color patches per video. We select a patch-size equal to 256×256 pixels as suggested in [1].

As for the audio, we extract the LMS based on the default parameters purposely designed for the VGGish network [24]. The investigated frequency range spans from 125 Hz to 7500 Hz; we exploit a sampling rate of 16,000 Hz and a window length of 0.025 s with hop length of 0.010 s. We end up with an LMS consisting of $H_{a}$ temporal samples and 64 Mel bins. Notice that the number of rows of LMS depends on the temporal length of the audio content, while the 64 Mel bins belong to the default parameters required by VGGish. Furthermore, after some preliminary experiments on how the exploited frequency range influences the classification performance, we propose to expand the investigated frequency range from 125 Hz to 20,000 Hz, changing the sampling rate to 44,100 Hz. Being that the investigated spectrum is enlarged by almost three times, we consider also three times as much the amount of Mel bins for computing the LMS. Therefore, we end up with an LMS with 192 Mel bins. In both the two situations, we randomly extract $N_{P_{a}}=500$ patches per LMS. As regards $H_{P_{a}}$ (i.e., the temporal dimension associated with the audio patches), we exploit the default parameter required by VGGish, i.e., 96 temporal bins. Thus, in the former scenario, the audio patch size is 96×64; in the latter one, the audio patch size is 96×192.

### 5.2. Network Setup and Training

As reported in Section 4.3, we always employ the EfficientNetB0 architecture for processing visual patches. On the contrary, we can use both VGGish and EfficientNetB0 architectures for processing the audio patches. Furthermore, the LMS can be calculated either on a reduced frequency range purposely designed for being processed by VGGish, or on an expanded range. In light of these considerations, we can work with three different network configurations per multi-modal method:

- Configuration EV, which uses EfficientNetB0 for processing visual patches and VGGish for audio patches, considering the default audio frequency range required by VGGish (i.e., 64 Mel bins);
- Configuration EE${}_{64}$, which uses EfficientNetB0 for both visual and audio patches, considering the same audio frequency range required by VGGish (i.e., 64 Mel bins);
- Configuration EE${}_{192}$, which uses EfficientNetB0 for both visual and audio patches, considering an expanded audio frequency range (i.e., 192 Mel bins).

Following a common procedure applied in CNN training, we initialize the EfficientNetB0 weights, using those trained on ImageNet database [45], while we initialize the VGGish ones using those trained on the AudioSet database [46]. We initialize in the same way also the weights of the EfficientNetB0 and of the VGGish networks that are part of the multi-input CNNs in the Early Fusion methodology. All CNNs are trained using the Cross-Entropy Loss and Adam optimizer with default parameters. The learning rate is initialized to 0.001 and is decreased by a factor of 10 whenever the validation loss does not improve for 10 epochs. We train the networks for at most 50 epochs, and training is stopped if the validation loss does not decrease for more than 20 epochs. The model providing the best validation loss is selected.

Concerning the dataset split policy, we always keep 80% of the video sequences of each device for the training phase (further divided in 85–15% for training set and validation set, respectively), leaving the remaining 20% to the evaluation set. All tests were run on a workstation equipped with one Intel^®^ Xeon E5-2687W v4 (48 Cores @3 GHz), RAM 252 GB, one TITAN V (5120 CUDA Cores @1455 MHz), 12 GB, running Ubuntu 20.04.2. We resort to Pytorch [47] as the Deep Learning framework.

### 5.3. Evaluation Metrics

To evaluate the goodness of the system in classifying video sequences we use confusion matrices, where rows and columns are associated with the smartphone models under analysis. The value at position (*i*, *j*) represents the probability that a patch of a video recorded by the *i*-th model is classified as a patch of a video recorded by the *j*-th model. The more effective the method, the more the confusion matrix tends to be diagonal. In particular, we evaluate results by means of the achieved balanced classification accuracy. These metrics can be computed as the average of the values lying on the confusion matrix diagonal.

### 5.4. Mono-Modal Results

In order to provide a baseline comparison with our proposed multi-modal attribution, we start showing the results achieved in the case of standard mono-modal attribution on the same dataset. Specifically, for both visual-based and audio-based attributions, we select the networks’ configuration achieving the average highest accuracy. In doing so, we select the EfficientNetB0 network for evaluating visual patches and the VGGish architecture for the audio ones, i.e., the networks’ configuration defined as EV.

**Figure 7:**
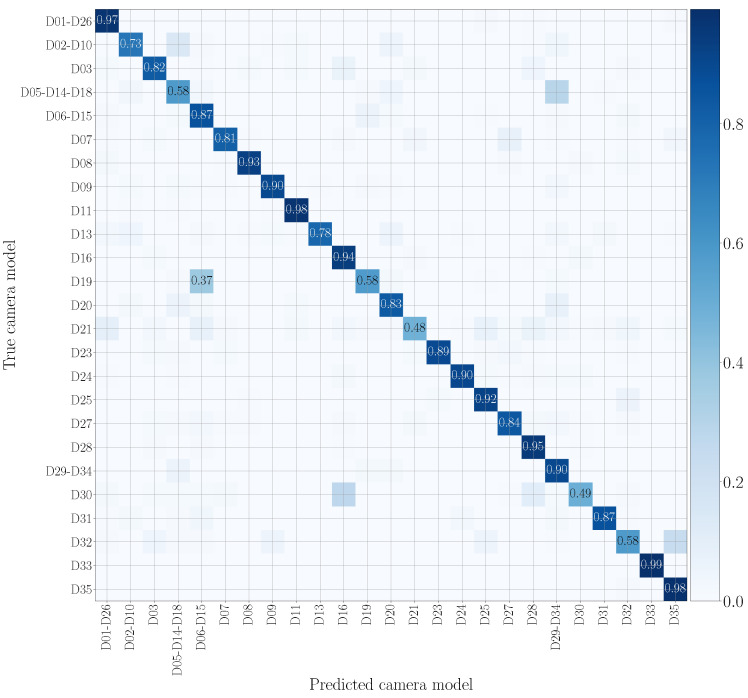
Confusion matrix achieved by mono-modal camera model identification exploiting visual patches only. We report results by training and testing on the native video set, and we only show the numbers which exceed 0.3. Device nomenclature is that of [44].

**Figure 8:**
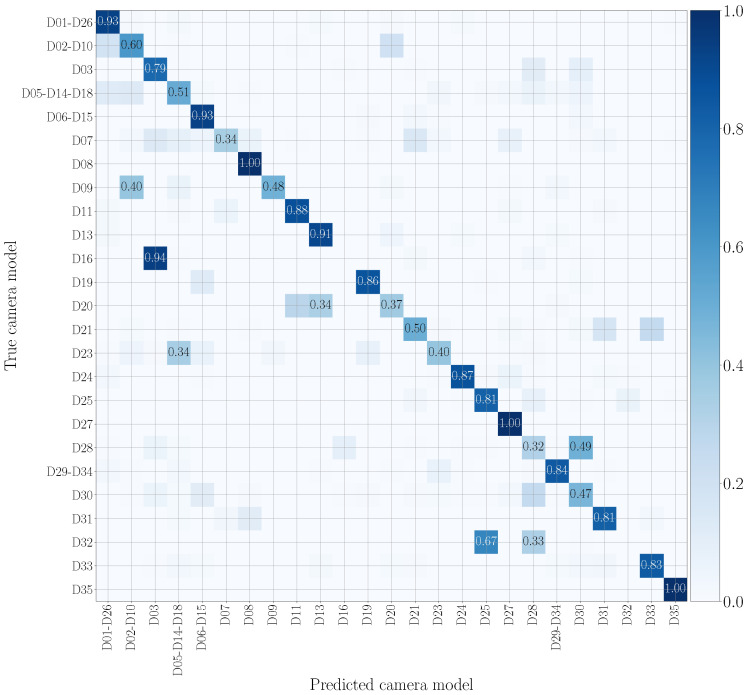
Confusion matrix achieved by mono-modal camera model identification exploiting audio patches only. We report results by training and testing on the native video set, and we only show the numbers which exceed 0.3. Device nomenclature is that of [44].

We report in Figure 7 and Figure 8 the confusion matrices obtained in the mono-modal scenarios, considering only visual patches or audio patches of native video sequences, respectively. As previously specified in Section 5.1, we group devices of the same camera model, such as D05, D14 and D18 (using the Vision dataset nomenclature), which are different instances of the Apple iPhone 5c model. It is worth noticing that there is some uncertainty in classification, especially in the second scenario. Nonetheless, as regards the visual mono-modal approach (see Figure 7), mismatches in classification only appear between very similar camera models, e.g., Apple iPhone 6 Plus (D19) is sometimes confused with Apple iPhone 6 (D06-D15). For what concerns the audio counterpart (see Figure 8), the classification errors are more distributed and may also occur between models of different vendors, e.g., OnePlus A3003 (D32) can be confused with Huawei P8 GRA-L09 (D28), and Asus Zenfone 2 Laser (D23) can be confused with Apple iPhone 5c (D05-D14-D18).

Having available the compressed versions of the videos with WhatsApp and YouTube algorithms, we investigate further by evaluating the cross test results, i.e., scenarios in which the data being tested have different characteristics than the training ones. For instance, we evaluate the achieved accuracy in testing WhatsApp video sequences by exploiting a network trained on native or YouTube compressed data. Table 1 shows the accuracy of cross tests and non-cross tests in both visual and audio modalities. We achieve the highest accuracy on the visual patches (82%) in the non-cross test configuration by working with native video sequences. Both the two mono-modal methods report similar performance on the WhatsApp and YouTube data in non-cross tests. Overall, we can leverage the visual content to achieve a better or comparable non-cross test performance.

Not surprisingly, the cross test results are worse than the non-cross test results, especially those including data from WhatsApp. In particular, we focus on the setup in which we train on native sequences and test on videos passed through WhatsApp and YouTube (see the first row of Table 1). Indeed, this represents a realistic scenario in which the forensics analyst has only available original data, but must investigate videos coming from social networks. The audio-based method is actually the best performing solution, outperforming its visual counterpart by almost 20% accuracy points. We think this superior performance may be due to a lighter compression applied by social media to the audio content with respect to the visual content. Since the audio content requires considerably less storage space than video frames, the audio track might undergo reduced compression operations, thus reporting weaker compression artifacts than those occurring in video frames. Therefore, the network trained on native audios can be better representative of WhatsApp and YouTube audio with respect to the network trained only on native visual content and tested on social network visual patches.

**Figure 9:**
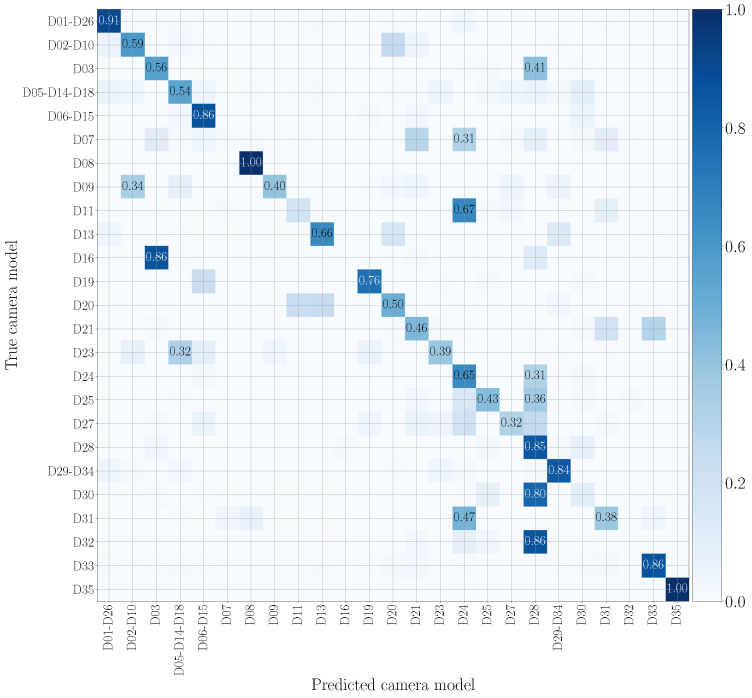
Confusion matrix achieved by mono-modal camera model identification, exploiting audio patches only. We report results by training on the native video set and testing on the WhatsApp set, and we only show the numbers that exceed 0.3. The device nomenclature is that of [44].

**Figure 10:**
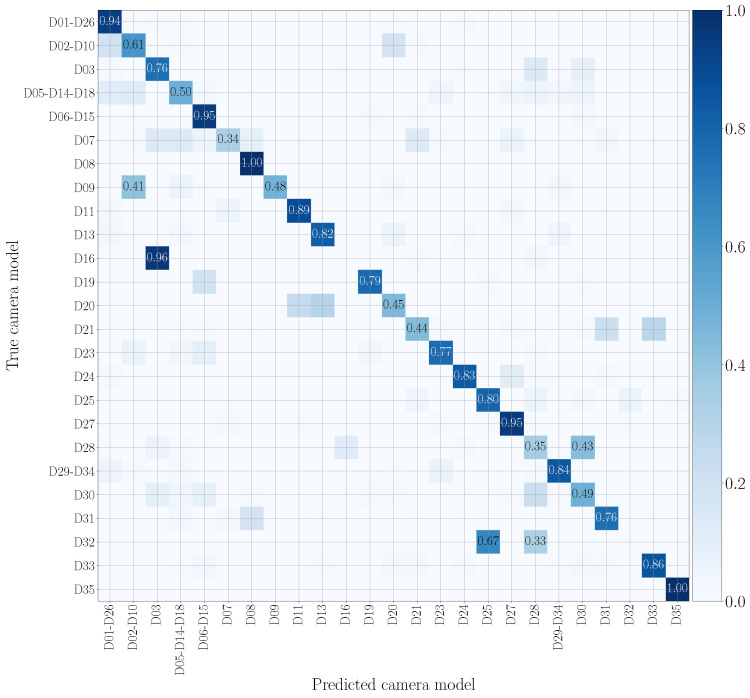
Confusion matrix achieved by mono-modal camera model identification, exploiting audio patches only. We report results by training on the native video set and testing on the YouTube set, and we only show the numbers which exceed 0.3. The device nomenclature is that of [44].

Figure 9 and Figure 10 draw the confusion matrices achieved in cross test scenarios by training on original audio patches and testing on WhatsApp and YouTube audio patches, respectively. WhatsApp data (see Figure 9) are the most challenging to model, and many camera models are confused with others of different vendors. This may be due to the compression operations performed by WhatsApp, which are more significant than those of YouTube, making classification more difficult. On the contrary, on YouTube data (see Figure 10), misclassifications mostly occur on models from the same brand, e.g., Huawei P9 Lite VNS-L31 (D16) is confused with Huawei P9 EVA-L09 (D03), and OnePlus A3003 (D32) is sometimes confused with OnePlus A3000 (D25).

### 5.5. Multi-Modal Results

As seen in Section 5.2, we can work with three different network configurations per multi-modal method: configuration EV (i.e., EfficientNetB0 for visual patches and VGGish for audio patches), configuration EE${}_{64}$ (i.e., EfficientNetB0 for both visual and audio patches, considering an audio frequency range composed by 64 Mel bins as required by VGGish), and configuration EE${}_{192}$ (i.e., EfficientNetB0 for both visual and audio patches, considering an expanded audio frequency range).

**Figure 11:**
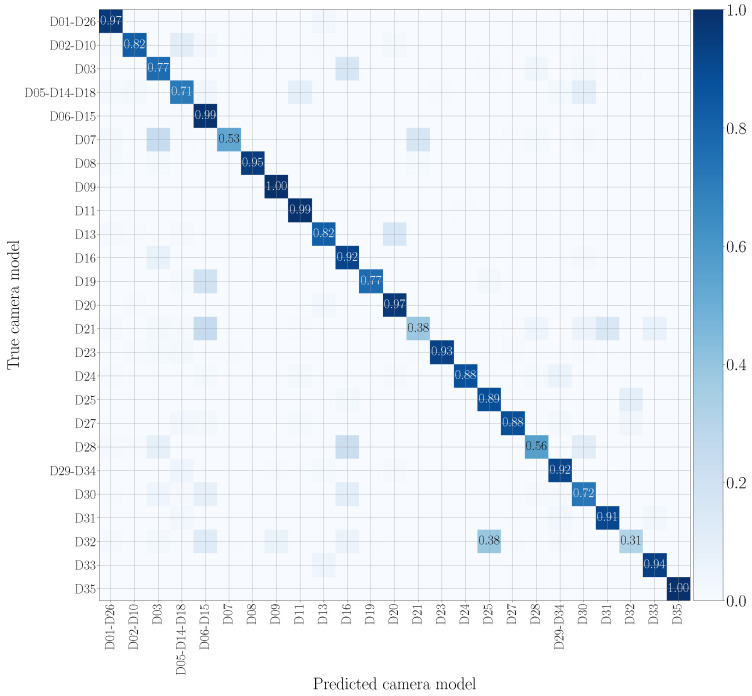
Confusion matrix achieved by multi-modal camera model identification exploiting Early Fusion EV. We report results by training and testing on the native video set, and we only show the numbers which exceed 0.3. Device nomenclature is that of [44].

**Figure 12:**
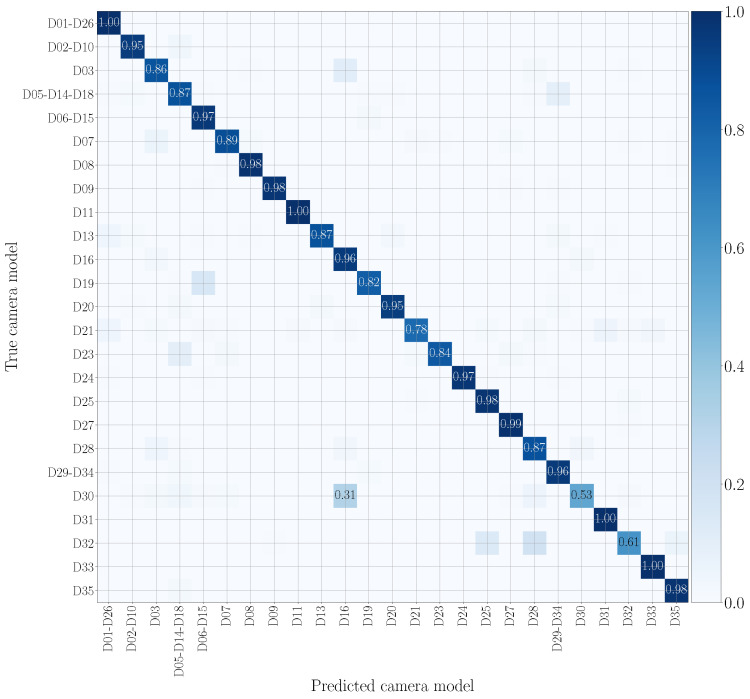
Confusion matrix achieved by multi-modal camera model identification exploiting Late Fusion EV. We report results by training and testing on the native video set, and we only show the numbers which exceed 0.3. Device nomenclature is that of [44].

In Figure 11 and Figure 12, we show the confusion matrices related to multi-modal camera model identification in a non-cross test scenario on the native video sequences. Specifically, Figure 11 refers to Early Fusion EV and Figure 12 to Late Fusion EV. In both cases, we consider the network’s configuration EV and the native video set to make a direct comparison with the mono-modal results previously reported in Figure 7 and Figure 8. The confusion matrix of Early Fusion has a similar behavior to the visual mono-modal results reported in Figure 7; the matrix approaches a diagonal style, but classification is not yet very effective. On the contrary, Late Fusion reports better performance; some misclassifications still occur (especially among models of the same vendor) but it shows a reduced error percentage.

Table 2 and Table 3 report the classification accuracy of Early Fusion and Late Fusion multi-modal methods, respectively. In particular, we investigate both non-cross and cross test scenarios, considering all the network configurations. As regards the non-cross tests on the native video set, the results obtained with multi-modal methods are always greater or comparable to those obtained with mono-modal methods. For instance, configuration EE${}_{192}$ achieves extremely high accuracy (up to 99%). In general, we obtain substantially better results also in the other non-cross tests, including YouTube and WhatsApp. For example, configuration EE${}_{192}$ always exceeds 91% accuracy on WhatsApp and 95% on YouTube.

Cross tests, including native and YouTube video sequences, follow this trend as well. On the other hand, cross tests on WhatsApp do not always significantly outperform the results achieved by mono-modal methods, being often comparable or superior.

**Figure 13:**
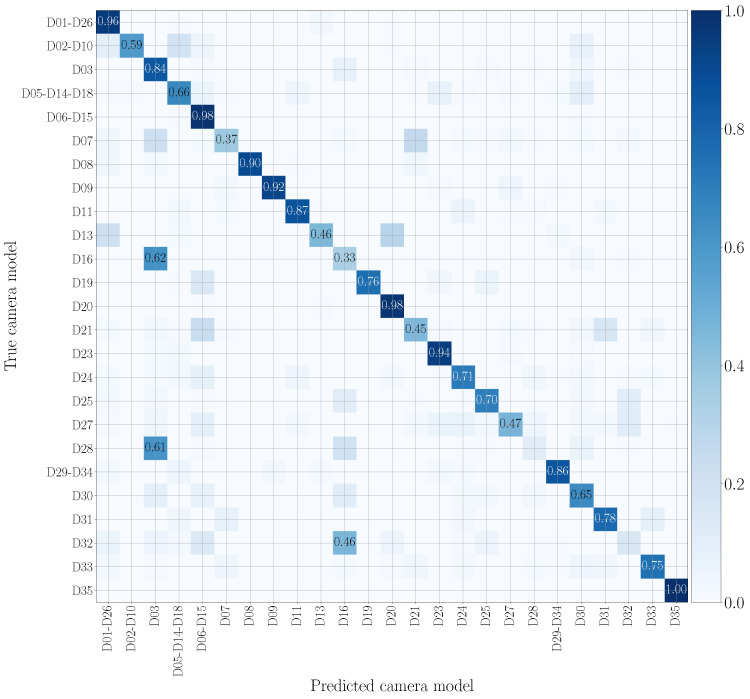
Confusion matrix achieved by multi-modal camera model identification exploiting Early Fusion EV. We report results by training on the native video set and testing on WhatsApp videos, and we only show the numbers that exceed 0.3. Device nomenclature is that of [44].

In particular, as was previously done for the mono-modal problem, we investigate the challenging scenario in which the training set consists of native video sequences, and testing data are picked from social media platforms (see the first row of Table 2 and Table 3). In this scenario, for WhatsApp, the proposed multi-modal methodologies achieve the best results with the Early Fusion EV configuration, outperforming the highest mono-modal accuracy by more than 15%. Interestingly, it is worth noticing that Early Fusion EV is the configuration that achieves the lowest non-cross test accuracy if compared to the remaining options. We think that a reduced overfitting on the training native set enables better results’ generalization also on testing data, which show quite different characteristics than training ones, WhatsApp videos being an example. Figure 13 depicts the confusion matrix corresponding to Early Fusion EV in the analyzed cross-test scenario. Contrarily to Figure 9 (which shows the confusion matrix for the same cross test scenario in the mono-modal setup), few misclassifications mainly occur among same-brand models.

**Figure 14:**
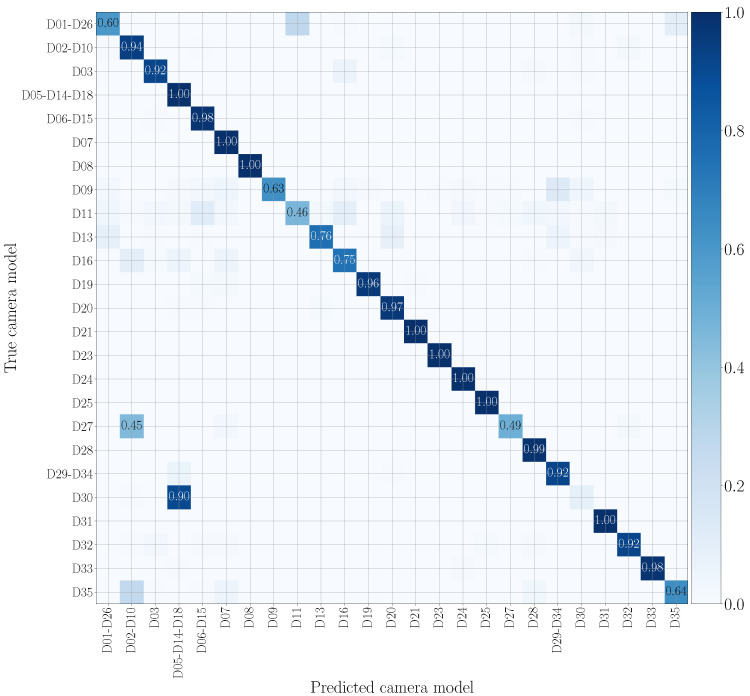
Confusion matrix achieved by multi-modal camera model identification exploiting Late Fusion EE${}_{192}$. We report results by training on the native video set and testing on YouTube videos, and we only show the numbers which exceed 0.3. Device nomenclature is that of [44].

Cross-test performance, by training on the native set and testing on YouTube, always exceeds that achieved by mono-modal methods. More specifically, Late Fusion EE${}_{192}$ outperforms the best mono-modal accuracy by 17%. In general, YouTube data are less prone to classification errors than WhatsApp. We are convinced that this is due to the weaker compression operations applied by YouTube, compared to WhatsApp, which render YouTube data more similar to the native ones. To provide an example, Figure 14 depicts the confusion matrix of Late Fusion EE${}_{192}$ in the analyzed cross-test scenario. Notice the diagonal behavior of the matrix; however, misclassifications sometimes occur among models of different vendors.

Comparing the two proposed multi-modal methods, Late Fusion always outperforms Early Fusion in non-cross tests scenarios. Nonetheless, the cross-test results show comparable accuracy between the two methods, and, on average, both the proposed methodologies report valid performance. Based on the scenario of our interest, we can prefer one proposed method over the other.

As for the comparison between the three networks’ configurations, EE${}_{192}$ obtains the best results in all non-cross-test scenarios for both the two proposed fusion methodologies. This consideration is valid for cross tests as well, considering data from the native and YouTube sets. However, when evaluating the cross test results with highly compressed data, such as those of WhatsApp, this is the configuration that works worst.

In general, we believe that the Late Fusion methodology associated with the EE${}_{192}$ configuration can be chosen as the best-preferred strategy among all the others. Indeed, it always reports the highest accuracy in both non-cross and cross test scenarios when dealing with native and YouTube video sequences. Cross-test results, including WhatsApp data, are comparable to the other two configurations, even if slightly worse. This lower performance can be attributable to the fact that, in this configuration, the trained CNNs adapt very well to the data seen in the training phase (i.e., visual and audio patches selected from native or YouTube video sequences), thus resulting in being less general and being more sensitive to significant data compression, such as that applied by WhatsApp.

## 6. Conclusions and Future Works

This paper proposes a novel multi-modal methodology for closed set camera model identification related to digital video sequences. In a nutshell, we propose to determine the smartphone model used to acquire a query video by exploiting both visual and audio information from the video itself. The devised methodology is based on CNNs capable of classifying videos by extracting suitable features from their visual and audio content. Given a video, as visual content, we use patches cropped from its video frames, while as audio content, we use patches cropped from the Log-Mel Spectrogram of its audio track.

We propose two multi-modal camera model identification approaches: in the Late Fusion method, we combine the scores individually obtained from two mono-modal networks (one working with visual patches and the other with audio patches) to classify the query video; in the Early Fusion method, we build one multi-input network and feed it with visual/audio patch pairs extracted from the query video. For each methodology, we compare three different networks’ configurations, exploiting distinct architectures and data pre-processing.

We evaluate our experimental campaign over video sequences selected from the Vision dataset. The videos on which we experiment are not only the original native ones, i.e., those directly acquired by the smartphone camera. We also use videos compressed by the WhatsApp and YouTube algorithms so as to explore many different training and testing configurations as well as to simulate realistic scenarios in which we have to classify data compressed through internet services (e.g., social media, and upload sites). Moreover, we compare our proposed multi-modal methodologies with a mono-modal attribution strategy selected as the baseline.

The achieved results show that the proposed multi-modal methods are significantly more effective than standard mono-modal methods; on average, the Late Fusion approach reports the best results. In general, we can correctly identify native and YouTube video sequences with accuracy of up to 99%. WhatsApp videos are yet the most challenging to model, probably due to the massive data compression applied. This opens the door to future challenges and improvements focused on identifying the source camera model on video sequences shared (or re-shared multiple times) on social media. Furthermore, it is worth noticing that the proposed multi-modal strategies could be straightforwardly extended to potential situations, including more data modalities (i.e., more than two). The Late Fusion methodology would only require separately training one CNN per modality, while the Early Fusion methodology would require training one multi-input CNN with a number of inputs equal to the available modalities.

## Figures and Tables

**Table 1:** Classification accuracy of mono-modal methods as a function of training/testing sets. In bold is the highest achieved classification accuracy.

-	Visual—EfficientNetB0			Audio—VGGish		
**Testing Set →**	**Native**	**WhatsApp**	**YouTube**	**Native**	**WhatsApp**	**YouTube**
**Training Set ↓**						
**Native**	**0.8202**	0.3579	0.4869	0.6578	0.5304	0.6654
**WhatsApp**	0.5599	0.6739	0.5158	0.5028	0.6757	0.5245
**YouTube**	0.7271	0.5531	0.7404	0.6954	0.5924	0.7010

**Table 2:** Classification accuracy of Early Fusion as a function of training/testing sets. In bold is the highest achieved accuracy in non-cross test scenarios.

-	Early Fusion EV			Early Fusion EE${}_{64}$			Early Fusion EE${}_{192}$		
**Testing Set →**	**Native**	**WhatsApp**	**YouTube**	**Native**	**WhatsApp**	**YouTube**	**Native**	**WhatsApp**	**YouTube**
**Training Set ↓**									
**Native**	0.8210	0.6879	0.7784	0.8396	0.6120	0.7956	**0.9598**	0.1795	0.7968
**WhatsApp**	0.5810	0.7519	0.5766	0.5930	0.8076	0.5873	0.5091	**0.9120**	0.4954
**YouTube**	0.7548	0.6212	0.7590	0.8071	0.6903	0.8090	0.8731	0.4146	**0.9513**

**Table 3:** Classification accuracy of Late Fusion as a function of training/testing sets. In bold, the highest achieved accuracy in non-cross test scenarios.

-	Late Fusion EV			Late Fusion EE${}_{64}$			Late Fusion EE${}_{192}$		
**Testing Set →**	**Native**	**WhatsApp**	**YouTube**	**Native**	**WhatsApp**	**YouTube**	**Native**	**WhatsApp**	**YouTube**
**Training Set ↓**									
**Native**	0.9039	0.5960	0.7069	0.8945	0.6020	0.8039	**0.9900**	0.4544	0.8389
**WhatsApp**	0.6413	0.7610	0.6368	0.6262	0.8198	0.6208	0.5703	**0.9163**	0.5602
**YouTube**	0.8163	0.6595	0.8274	0.8321	0.6976	0.8390	0.9172	0.4957	**0.9519**
